# Women are worse off in developing and recovering from temporomandibular disorder symptoms

**DOI:** 10.1038/s41598-025-86502-0

**Published:** 2025-02-08

**Authors:** Anna Lövgren, Simon Vallin, Birgitta Häggman-Henrikson, Flavia P. Kapos, Christopher C. Peck, Corine M. Visscher, Per Liv

**Affiliations:** 1 Department of Odontology/Orofacial Pain and Jaw Function, Faculty of Medicine, Umeå University, 901 87 Umeå, Sweden; 2 Department of Orofacial Pain and Jaw Function, Malmö University, Malmö, Sweden; 3 Department of Orthopaedic Surgery & Duke Clinical Research Institute, Duke University School of Medicine, Durham, USA; 4 Faculty of Dentistry, National University of Singapore, Singapore, Singapore; 5 Department of Orofacial Pain and Dysfunction, Academic Centre for Dentistry Amsterdam (ACTA), University of Amsterdam and Vrije Universiteit Amsterdam, Amsterdam, The Netherlands; 6 Section of Sustainable Health, Department of Public Health and Clinical Medicine, Umeå University, Umeå, Sweden

**Keywords:** Decision-making, Epidemiology, Facial pain, Temporomandibular joint dysfunction syndrome, Neuroscience, Health care, Medical research, Risk factors

## Abstract

Decision-making for temporomandibular disorders (TMDs) is reported being a clinical challenge, partly due to uncertainities in assessment of long-term prognosis. Therefore, our aim was to explore variations over time in TMD symptoms and possible sex or age differences. In this cohort study, data were prospectively collected 2010–2017 from the general population in Västerbotten, Northern Sweden. Adults were eligible if they had undergone at least two routine dental check-ups that included screening for TMDs (3Q/TMD) from which states were defined as absence or presence of TMD pain and/or jaw catching/locking. The rate of transitions was estimated between TMD states within a time span of one year. A total of 94,769 individuals were included (49.9% women) with 205,684 repeated visits and 9,006 state transitions recorded over the 8-year period. Compared to men, women had higher rates of transitions from no TMDs to any TMD symptoms. Furthermore, women had a lower rate of transition from TMD pain only to no TMDs. The finding of a poorer prognosis in women, as well as previously reported potential gender differences in pain perception and reporting, reinforces that gender differences should be accounted for in the treatment planning stage for patients with onset of TMDs.

**Supplementary Information:**

The online version contains supplementary material available at 10.1038/s41598-025-86502-0.

## Introduction

Temporomandibular disorders (TMDs) include pain in the oral and craniofacial region, and/or functional limitations such as catching or locking of the jaw^1–3^. With a prevalence of approximately ten percent in the general population, orofacial pain, including TMD pain, is one of the most common chronic pains^4,5^. There are however other clinical signs related to TMDs, for example joint sounds, restricted mouth opening and pain on palpation. As such, clinical signs of TMDs can be even more common than the symptoms evaluated in the present study.

The prevalence of TMDs increases during adolescence, is highest among working-aged individuals, is less frequent in the older adults, and is higher in women across all age groups^5–7^. Despite similar TMD pain incidence for women and men^8^, women can report longer pain duration (which may account for their higher pain prevalence), higher pain intensity and impact, more clinical symptoms, and more care-seeking^9^. Interestingly, we recently showed an increasing prevalence of orofacial pain in the general population that was more so in women than in men, which indicates that the prevalence gap is widening^10^. However, the reasons for these population and especially female increases are unknown.

At the individual level, the trajectories for TMD onset, persistence, or recovery vary significantly from person to person. While psychosocial factors such as somatic symptoms can increase the risk for TMD pain onset^11,12^, and negative pain expectations can predict long-term outcomes^13^, there are limited data on the natural course of the development and maintenance of pain and functional limitations, including differences between men and women over extended periods of time. This, together with the lack of longitudinal data from the general population, has resulted in a gap of knowledge on TMD symptom variations over time, and whether such variations differ by sex and age.

Given the burden of TMD symptoms on societal and individual levels^11^, early identification along with management in line with evidence-based recommendations have been suggested as key factors in improving long-term outcomes of affected individuals^14^. Nevertheless, a significant number of patients with TMD are currently not receiving optimal healthcare management. General dental practitioners encounter patients with TMD on a regular basis and are therefore critically important in the management of such disorders. However, since dentists report uncertainties in their clinical decision-making for TMDs, there is a need for improving the care journey for affected patients^15^. In this context, improved understanding of TMD variations can improve clinical guidelines for long-term personalized TMD management strategies. Therefore, our aim was to explore variations in two TMD-related symptoms; TMD pain and jaw catching/locking, in a population-based sample of adults, and whether such variations were influenced by sex or age.

## Methods

### Study setting and sample

**Fig. 1:**
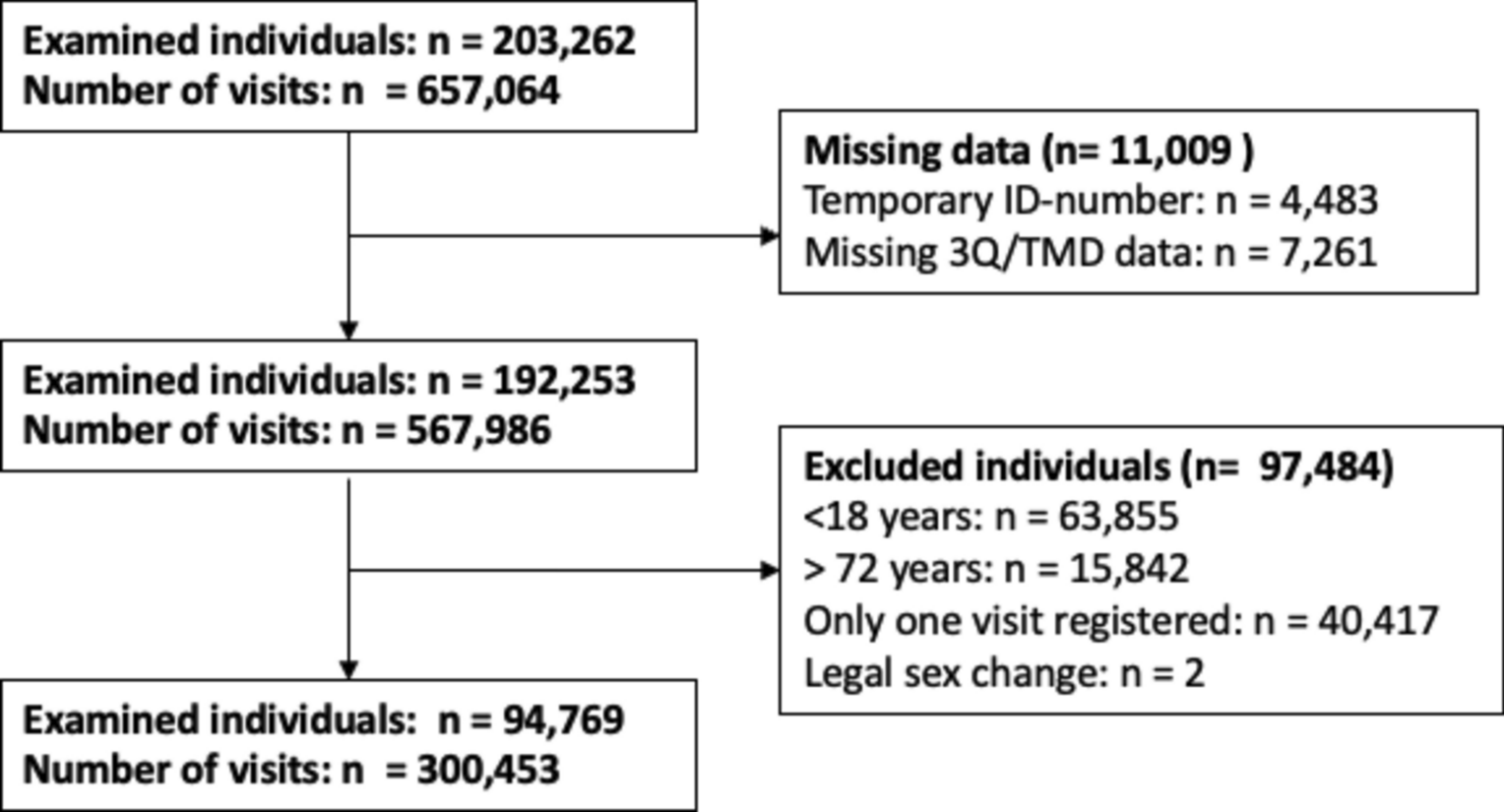
Flow chart of the inclusion process and study sample.

This study was based on data collected May 2010–December 2017 in the county of Västerbotten, Northern Sweden. Sweden’s universal dental care subsidized by the national government. All citizens are provided subsidies for general and special dental care (oral-systemic health issues), with a focus on preventive care and a safety net for high dental care costs. Consequently, approximately 80% of the population undergo regular dental check-ups, and in Västerbotten more than 50% of adults do so within the Public Dental Health service. In the present study, individuals were eligible for inclusion if they were 18–72 years of age, had undergone at least two routine dental check-ups in the Public Dental Health service, and at their appointments had completed a digital health history. Data on sex and age were extracted from the personal identity numbers in the dental records. Temporary residents were excluded due to difficulties with follow-up over time. Furthermore, two individuals who legally changed their sex during the study period were excluded. Figure 1 shows a flowchart of inclusion and exclusion number of individuals and number of visits. The study protocol was approved by the Swedish Ethical Review Authority (ref no 2019-04534).

### Outcome measures

Data on self-reported TMD pain and jaw function limitations were retrieved from the digital health history based on individual responses to the following TMD screening questions (3Q/TMD^16^).

Q1: Do you have pain in your temple, face, jaw, or jaw joint, once a week or more?

Q2: Do you have pain when you open your mouth or chew once a week or more?

Q3: Does your jaw lock or become stuck once a week or more?

All questions are answered with a “yes” or a “no”.

**Fig. 2:**
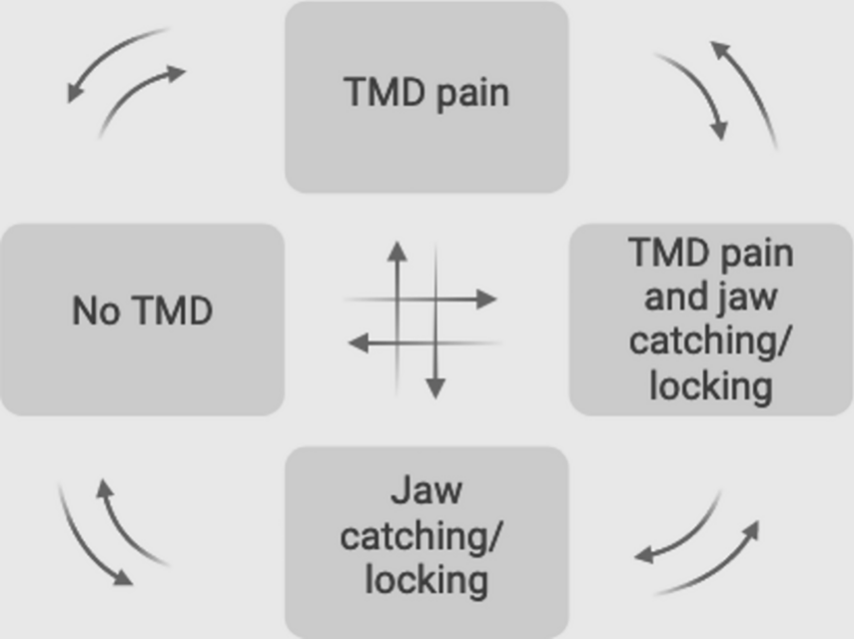
Schematic illustration of possible transition states analyzed within the data (N = 94,769).

The two questions on pain (Q1 and Q2) have been validated for the most frequent TMD-pain diagnoses^16,17^, and the question on function (Q3) for identifying jaw function limitations^18^. Thus, an affirmative answer to Q1 and/or Q2 was categorized as TMD pain, whereas an affirmative answer to Q3 was categorized as jaw catching/locking. From these responses, at each visit an individual was classified in one of four states (Fig. 2):

- no TMD (i.e. no pain or jaw catching/locking)
- TMD pain only (no jaw catching/locking)
- jaw catching/locking only (no pain)
- TMD pain and jaw catching/locking

Each individual’s TMD state was classified into a single TMD state per calendar year of the visit. For those who had more than one visit in a single calendar year, affirmative answers to the 3Q/TMD in any of these visits were considered to determine the individual’s TMD state for that year.

#### Statistical methods

Descriptive statistics for the participant group overall, and for women and men subgroups, were determined and included participant demographics, number of dental visits, TMD state, and the number of recorded transitions between the different states. A Markov multistate model, with sex as explanatory variable while using visit year as a continuous variable of time, was used to analyze time to transitions between the four mutually exclusive states as indications of variations in TMD symptoms. The multistate model can be considered an extension of the traditional survival analysis (i.e., Cox proportional hazards model) that allows estimation of the probabilities of moving between multiple states instead of only from one state to another or experiencing a single event. Each individual was assumed to be at risk of transitioning between states from their first until their last recorded visit. The model was estimated under the assumption that the individuals remained in the same state until a visit recorded a transition to a different state. The rate of transitions within each sex and age group was evaluated using stratified multistate models without explanatory variables. From each of the stratified models, the probability of all possible transitions between the states within a time span of one year was estimated, along with a 95% confidence interval.

Statistical analyses were performed using R^19^. The multistate models were fitted using the *msm* package^20^.

The study was approved by the Regional Ethical Board at Umeå University (ref no 2012-331-31M and 2019-04534) and performed in accordance with the Declaration of Helsinki. Due to the retrospective nature of the study, the Regional Ethical Board at Umeå University waived the need of obtaining informed consent. The STROBE statement was followed for reporting^21^.

## Results

In total, 94,769 individuals (49.9% women), contributing with 400,150 person-years in total, were included in the analysis (Table 1). Of these, 2,513 (2.7%) had more than one visit recorded during a single year. Most individuals had between two and four visits at least one year apart, resulting in one to three possible state transitions. The number of visits per individual was equally distributed between women and men, and increased slightly with age (mean number of visits 3.0 and 3.4 for 18–29 year olds and 60–72 year olds, respectively). The period prevalence of *two reported symptoms once a week or more,* TMD, i.e., the proportion of individuals who reported any TMD at least once between May 2010 and December 2017, was 11.3% (TMD pain: 9.0% and jaw catching/locking: 4.6%). At the individual level, 8.0% reported transitions in *TMD pain* and/or *jaw catching/locking* states.

**Table 1:** Participant characteristics in the study sample.

	Overall n = 94,769	Women n = 47,336	Men n = 47,433
Age (yrs, IQR)	37 (23, 52)	36 (23, 52)	37 (23, 52)
Number of visits			
2	30,252 (32%)	15,206 (32%)	15,046 (32%)
3	31,782 (34%)	15,900 (34%)	15,882 (33%)
4	21,899 (23%)	10,863 (23%)	11,036 (23%)
5	8,486 (9.0%)	4,165 (8.8%)	4,321 (9.1%)
6	1,914 (2.0%)	970 (2.0%)	944 (2.0%)
7	395 (0.4%)	208 (0.4%)	187 (0.4%)
8	41 (< 0.1%)	24 (< 0.1%)	17 (< 0.1%)
Number of visits with reported TMD pain			
0	86,213 (91%)	41,157 (87%)	45,056 (95%)
1	3,583 (3.8%)	2,475 (5.2%)	1,108 (2.3%)
2	2,653 (2.8%)	1,925 (4.1%)	728 (1.5%)
3	1,419 (1.5%)	1,077 (2.3%)	342 (0.7%)
4	633 (0.7%)	487 (1.0%)	146 (0.3%)
5	217 (0.2%)	173 (0.4%)	44 (< 0.1%)
6	41 (< 0.1%)	32 (< 0.1%)	9 (< 0.1%)
7	8 (< 0.1%)	8 (< 0.1%)	0 (0%)
8	2 (< 0.1%)	2 (< 0.1%)	0 (0%)
Number of visits with reported jaw catching/ locking			
0	90,454 (95%)	44,403 (94%)	46,051 (97%)
1	1,713 (1.8%)	1,165 (2.5%)	548 (1.2%)
2	1,402 (1.5%)	931 (2.0%)	471 (1.0%)
3	732 (0.8%)	514 (1.1%)	218 (0.5%)
4	342 (0.4%)	233 (0.5%)	109 (0.2%)
5	103 (0.1%)	68 (0.1%)	35 (< 0.1%)
6	15 (< 0.1%)	15 (< 0.1%)	0 (0%)
7	8 (< 0.1%)	7 (< 0.1%)	1 (< 0.1%)

Of the 205,684 available repeated visits, 9,006 (4.4%) state transitions were recorded (Table 2). The two most common state transitions were from *no TMD* to *TMD pain only,* followed by the transition from *TMD pain only* to *no TMD*.

**Table 2:**
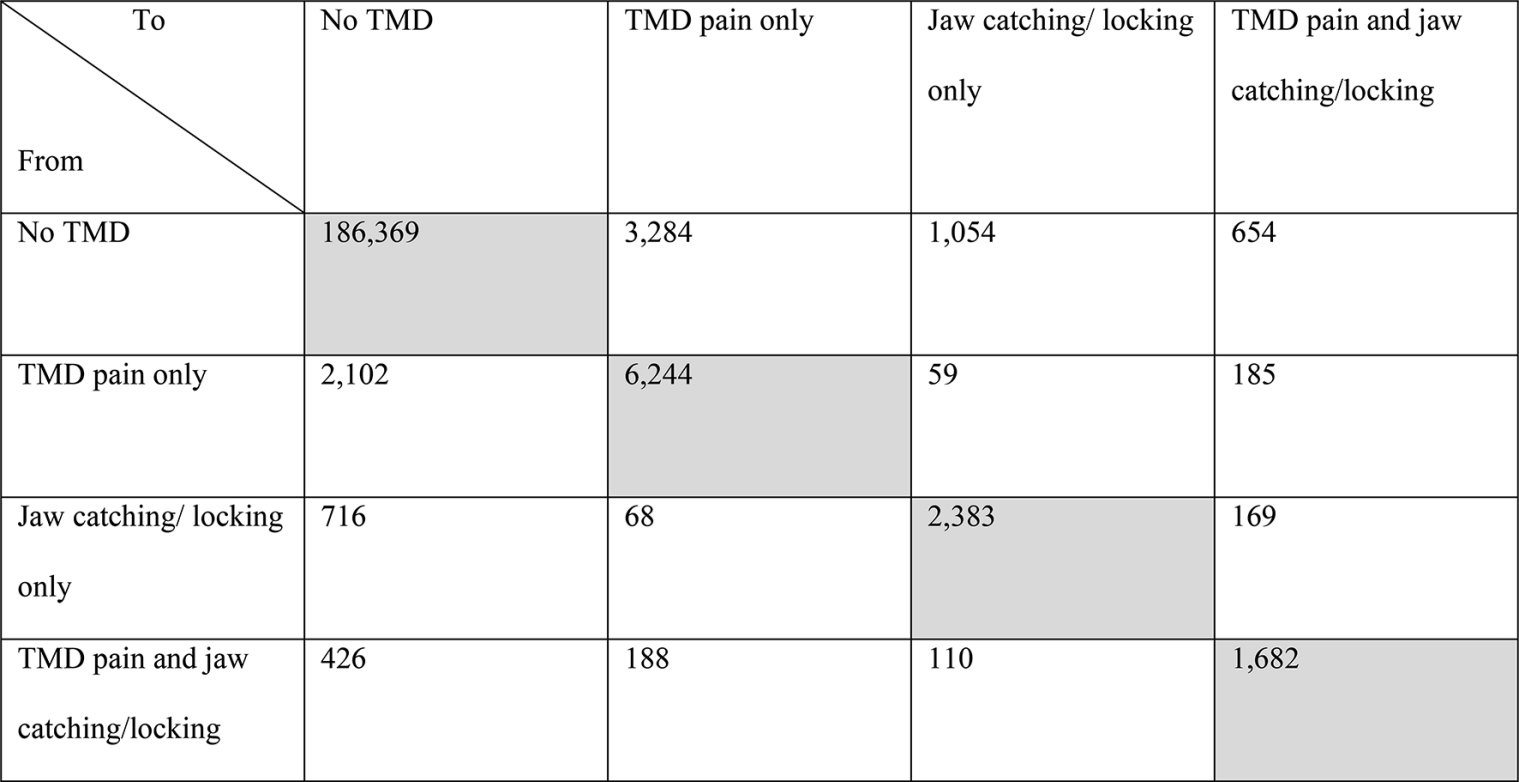
Recorded transitions between states among all participants (N = 94,769). Note that the diagonal of the table (grey cells) represents the number of visits where individuals remained in the same state.

Compared to men, women had higher rates of transitions from *no TMD* to all other three symptomatic states—*TMD pain only* (Hazard ratio (HR): 2.40, CI:2.22–2.59)*, jaw catching/locking only* (HR: 1.81, CI:1.59–2.07)*,* and *TMD pain and jaw catching/locking* (HR: 2.80, CI:2.28–3.43) (Table 3). Moreover, women had a higher rate of transition from *jaw catching/locking only* to *TMD pain and jaw catching/locking* (HR:1.62, CI:1.15–2.30). Furthermore, compared to men, women had a lower rate of transition from TMD *pain only* to *no TMD, (HR:* 0.83, CI: 0.75–0.91).

**Table 3:**
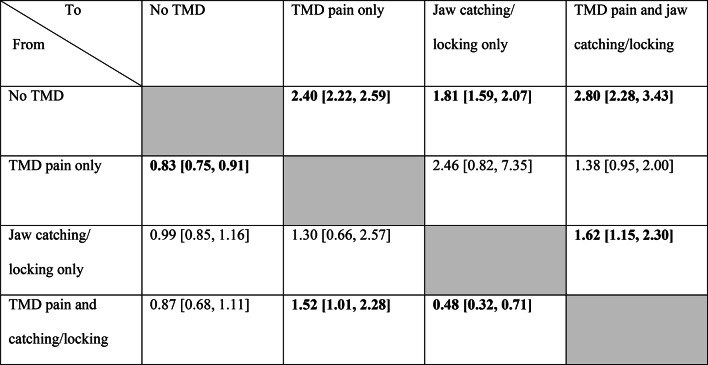
Hazard ratio with 95% confidence interval comparing the hazard of all transitions between women and men (reference). Bold text indicates significant differences at a 5% significant level.

**Fig. 3:**
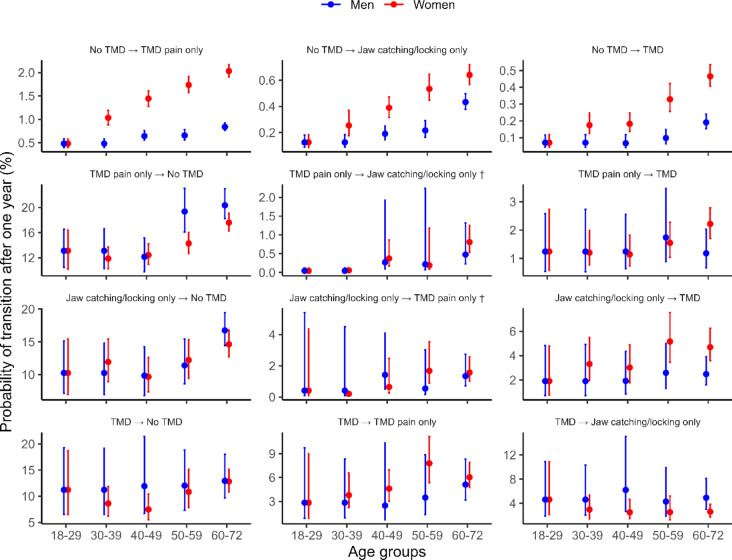
Probabilities of transitions in the study sample, stratified by sex and age. Note that the y-axis scale varies between different transitions and that y-axes does not start at zero to facilitate comparison between strata within the transitions. † For transitions between *TMD pain only* and *jaw catching/locking only* (both directions), the number of transitions was not sufficient to estimate meaningful confidence intervals, which are therefore omitted in the figure.

With increasing age, there were higher one-year risks of transitioning from *no TMD* to all other three states (Fig. 3). For all age groups over 30 years, women had a higher risk of onset of TMD symptoms (TMD pain and/or jaw catching/locking) than men. For both women and men, there was a higher probability to recover from *TMD pain only* to *no TMD* for groups over 50 years. In addition, there was a higher probability of recovering from *jaw catching/locking only a*mong the 60–72 year olds*.*

## Discussion

The main finding from this longitudinal population-based study of Swedish adults attending routine dental check-up appointments in public dental health services, was that women reported transitions from *no TMD to TMD pain only* and to *jaw catching/locking* (with or without TMD pain) more frequently than men, especially from the age of 30 and onwards. Furthermore, men tended to recover more often than women, especially in relation to TMD pain alone.

Even though the low probability of transitioning into TMD states, our study demonstrates that despite TMD symptom variations over time, once you have a TMD you are more likely to develop a persistent condition. Our findings stress the chronic nature of TMDs since the probability of transitioning to *no TMD pain* and/or *jaw catching/locking* was low (10–20%) for all participants with women tending to recover less often, particularly from TMD pain only. Previous studies using convenience samples suggest that even though TMD symptoms tend to fluctuate over time^22^, the long-term prognosis, on average, is mainly described as positive or neutral, with an overall decrease in TMD pain intensity (and not necessarily complete resolution)^12^, and with no progression of intraarticular findings on imaging examinations^23^. A recent study on jaw pain fluctuations over 30 days suggested that cases with high impact pain were more likely to report clinically significant short-term fluctuations^24^. Since TMD pain in general population samples is more likely to be of low to moderate intensity^16^, the pain intensity may partly explain our study’s low probability of change over a one-year period. All in all, and in line with previous findings, variations in pain were more frequent when compared to jaw catching/locking.

In general, the prevalence of numerous pain conditions tends to increase with age^25^. This trend is even more pronounced in low back pain, the most common chronic pain location, where the prevalence has been shown to increase until approximately 80 years of age^26^. In contrast, for TMDs, the commonly reported inverted U-shaped curve illustrates how the prevalence of TMD gradually diminishes from age 60 onwards^27,28^. Previous studies suggest that the distribution of specific TMD diagnoses varies with age. Thus, painful symptoms such as arthralgia are more common in older adult patients, whereas disc displacement and muscle disorders are more common in younger patients^29^. These findings also align with a study on a patient sample in China^30^ that found that disc displacement was more common among patients in the younger group, whereas TMD pain was more common among individuals 45–65 years of age. In line with these findings, our results show that recovery from both TMD pain and jaw catching/ locking is more common from the age of 50 and onwards. The reason for such recovery is not known, but it has been speculated to be related to adaptation and acceptance, or the onset of other more severe conditions that dominate the individual’s attention. To date, most of the clinical decision-making regarding prognosis of TMDs in the general population relies on empirical evidence and observations. Our findings provide evidence on an expected positive development of TMD symptoms on the general population level–reassuring the individual on the benign nature of TMD that may have a positive impact on prognosis.

While there are more similarities than differences between women and men in skills and prevalences of diseases and illnesses^31,32^, this is not the case for pain prevalence^33,34^. In line with this, our study demonstrates that variations in symptoms over time also differ between men and women. Although the reasons for these differences by sex are still to be determined, they are likely not only related to biological differences, but are also influenced by gender perspectives that include expectations on behaviors such as pain reporting and care-seeking^35^. One such example is that an individual’s pain reporting varies depending on the examiner’s gender^36^. With a female examiner, women reported higher pain intensities, and with a male examiner, pain tolerances were increased for both women and men. These differences likely stem from gender normative behaviors rather than biological reasons as part of the important interpersonal aspects to be considered in pain assessment^37^. As a consequence, guidelines for experimental studies recommend reporting the participants’ and examiners’ gender (in addition to sex assigned at birth) due to its influence on pain reporting^38^. However, such risks of gender bias or differences are often overlooked in clinical guidelines and epidemiological studies on pain. Exploring pain reporting together with interpersonal aspects including sex and gender, and their impact on prevalence warrants further investigation.

At present, the management of individuals with TMDs is suboptimal for a number of reasons that include clinicians’ diagnostic uncertainties^39^, under-treatment, and prioritizing management of other dental conditions^40,41^. Furthermore, gender norms have also been identified as significant factors in biased decision-making particularly in the management of painful conditions^42,43^. For example, when compared to men, women remain on waiting lists longer^44^, are attributed psychological causes for their pain more frequently^35^, and are prescribed self-care more frequently as opposed to in-person clinical management^45^. Since prognosis is also influenced by management, gender bias (e.g., delays in women’s TMD treatment) might be one of the reasons behind the identified better prognosis in men when compared to women. Our findings suggest future studies should test, for example, other risk factors for state transitions and whether more frequent follow-ups in women could reduce both sex differences and overall burden of TMDs.

## Strengths and limitations

The present study included data from a large population sample based on validated screening questions for TMDs to assess TMD variations longitudinally over an 8-year period. The assumption was made that individuals remained within the same state until a visit with a state transition was recorded. This implies that there is a risk of confounding due to differences in visiting frequency (e.g. if women had a markedly higher visiting frequency than men, they would have had more opportunities for transitions, potentially leading to spurious differences in transition rates). However, in our study, the distribution of number of visits by sex was nearly identical for men and women which reduces the potential impact of this type of bias on our findings related to sex differences. The presented one-year probabilities of transitions between states have undoubtedly been affected by the frequency of dental visits; a population with more frequent dental visits would have more reported data from the screening questions, increasing the opportunities and likelihood for a state transition within a specific time-period. Therefore, the estimated probabilities should primarily be used for relative comparisons between the sex- and age groups, rather than being overly emphasized in terms of their absolute sizes. The low probability of recovery from any TMD in our study may have been influenced by participants self-selecting their dental appointment frequency. Thus, participants with more chronic TMDs may have sought treatment more frequently than those with acute or improving TMD symptoms. In the context of higher frequency of dental appointments, this may be related to patients’ concerns and anxiety about their complaints. This highlights the importance of a proper TMD examination and diagnosis to validate patients’ experiences and ease the negative effects from anxiety. Information about received treatment is an important factor in relation to variations in TMDs. However, this information was not available in this study sample. Future studies, also aiming to reveal causal relationships, should take this into account. Although we did not have individual data on gender identity, future studies should investigate how and to what extent the observed differences may be related to the social context of gender, or to biological variation by sex.

## Conclusion

Compared to men, women collectively experience TMD onset more frequently and recover less often, especially in relation to TMD pain alone. The finding of a poorer prognosis in women with pain, reinforces that sex and gender differences should be accounted for in the treatment planning stage for patients with the onset of TMDs. Given the sparse data available, research is needed to identify biopsychosocial determinants of sex and gender differences in TMD symptom variations to improve clinical decision-making because this may help reduce the burden of TMDs in the general population.

## Supplementary Information

Supplementary Information.

## Data Availability

The data that support the findings of this study are available from Region Västerbotten but restrictions apply to the availability of these data, which were used under license for the current study, and so are not publicly available. Data are however available from the corresponding authors upon reasonable request and with permission of Region Västerbotten.
