# Supplementary material for: Women are worse off in developing and recovering from temporomandibular disorder symptoms
Source: Sci Rep. 2025 Feb 8;15:4732. doi: 10.1038/s41598-025-86502-0 (PMC11807177; doi:10.1038/s41598-025-86502-0)
Supplement: Supplementary file 1 — Supplementary Information. [file 41598_2025_86502_MOESM1_ESM.docx]

Table S1. Recorded transitions between states among men (n=47,433). Note that the diagonal of the table (grey cells) represents the number of visits where individuals remained in the same state.

| To  From | No TMD | TMD pain only | Jaw catching/ locking only | TMD pain and jaw catching/locking |
| --- | --- | --- | --- | --- |
| No TMD | 97,513 | 1,000 | 398 | 175 |
| TMD pain only | 639 | 1,599 | 9 | 37 |
| Jaw catching/ locking only | 277 | 20 | 916 | 46 |
| TMD pain and jaw catching/locking | 109 | 30 | 42 | 353 |

Table S2. Recorded transitions between states among women (n=47,336). Note that the diagonal of the table (grey cells) represents the number of visits where individuals remained in the same state.

| To  From | No TMD | TMD pain only | Jaw catching/ locking only | TMD pain and jaw catching/locking |
| --- | --- | --- | --- | --- |
| No TMD | 88,856 | 2,284 | 656 | 470 |
| TMD pain only | 1,463 | 4,645 | 50 | 148 |
| Jaw catching/ locking only | 439 | 48 | 1467 | 123 |
| TMD pain and jaw catching/locking | 317 | 158 | 68 | 1,329 |
